# Drug repositioning for dengue haemorrhagic fever by integrating multiple omics analyses

**DOI:** 10.1038/s41598-018-36636-1

**Published:** 2019-01-24

**Authors:** Takayuki Amemiya, M. Michael Gromiha, Katsuhisa Horimoto, Kazuhiko Fukui

**Affiliations:** 1 Molecular Profiling Research Center for Drug Discovery (molprof), National Institute of Advanced Industrial Science and Technology (AIST), Tokyo, 135-0064 Japan; 2 Department of Biotechnology, Indian Institute of Technology Madras, Tamil Nadu, 600 036 India

## Abstract

To detect drug candidates for dengue haemorrhagic fever (DHF), we employed a computational drug repositioning method to perform an integrated multiple omics analysis based on transcriptomic, proteomic, and interactomic data. We identified 3,892 significant genes, 389 proteins, and 221 human proteins by transcriptomic analysis, proteomic analysis, and human–dengue virus protein–protein interactions, respectively. The drug candidates were selected using gene expression profiles for inverse drug–disease relationships compared with DHF patients and healthy controls as well as interactomic relationships between the signature proteins and chemical compounds. Integrating the results of the multiple omics analysis, we identified eight candidates for drug repositioning to treat DHF that targeted five proteins (ACTG1, CALR, ERC1, HSPA5, SYNE2) involved in human–dengue virus protein–protein interactions, and the signature proteins in the proteomic analysis mapped to significant pathways. Interestingly, five of these drug candidates, valparoic acid, sirolimus, resveratrol, vorinostat, and Y-27632, have been reported previously as effective treatments for flavivirus-induced diseases. The computational approach using multiple omics data for drug repositioning described in this study can be used effectively to identify novel drug candidates.

## Introduction

Mosquito-based diseases, such as malaria, dengue, and chikungunya, are life-threatening, so the development of vaccines and medicines for these diseases is of utmost importance for human health. Dengue is one of the most rapidly spreading mosquito-borne diseases worldwide, and its distinguishing features are bleeding and high fever. The dengue virus is a member of family Flaviviridae and has five antigenically distinct serotypes (dengue virus type 1 to 5). Dengue has an estimated annual incidence of about 100 million cases, resulting in about 500,000 yearly clinical cases of dengue haemorrhagic fever (DHF) syndrome, of which 5% are fatal^1–3^. DHF is characterized by vasculopathy, which results in sudden plasma leakage that reduces the blood volume and can result in hypovolemic shock, known as dengue shock syndrome. The World Health Organization has classified dengue infection as a neglected tropical disease. More than one billion people are affected by neglected tropical diseases annually, and these diseases cost developing economies billions of dollars every year. Despite the urgent need, so far, no effective antiviral agents have been identified for treating dengue infection and existing treatments are only supportive. Furthermore, no licensed vaccines against dengue infection are available. Previous attempts to develop drugs for DHF used structure-based and fragment-based approaches to modify existing potent antiviral agents^4–8^. Although both *in silico* and *in vitro* studies have reported several compounds as being dengue virus inhibitors, only chloroquine^9^, celgosivir^10^, and balapiravir^11^ progressed to clinical trial testing found in databases of clinical studies (ClinicalTrial.gov; https://clinicaltrials.gov/, and Clinical Trial Resister EU: https://www.clinicaltrialsregister.eu/). Unfortunately, none of these compounds produced satisfactory clinical trial results. Thus, there is still an urgent need to design better medication for treating dengue viral infection.

Traditional drug discovery takes enormous amounts of time, money, and effort to find a new drug. In addition to these high costs, the probability of a promising candidate molecule eventually becoming a US Food and Drug Administration (FDA)-approved drug is very low. These challenges and problems can be overcome by drug repositioning/repurposing, which is a drug discovery strategy that seeks to expand indications for approved drugs or to renew failed drugs. In this approach, the target drugs have already been tested for their effectiveness against other diseases or conditions and have been proven safe for human use; hence, the success rate in this technique is expected to be high. Approaches that are cost-effective are particularly important when working to discover innovative drug treatments for rare and/or neglected diseases, because typically less funding is available for these studies. Drug repurposing has been applied by several groups aiming to identify suitable therapeutic treatments for dengue infection. The methods used in these studies involved drug repositioning based on clinical knowledge about the reduction of dengue symptoms. The results supported repurposing prochlorperazine^12^, nordihydroguaiaretic acid^13^, minocycline^14^, doxycycline^15^, and amodiaquine^16^ for dengue infection. Additionally, Chen *et al*.^17^ investigated the repurposing of a library of pharmacologically active compounds (LOPAC^1280^) using a screening approach with Huh-7 cells and identified three compounds, N-desmethylclozapine, fluoxetine hydrochloride, and salmeterol xinafoate, as dengue virus inhibitors. However, none of these candidate dengue virus inhibitors have advanced to clinical trials in human.

Here, we applied a computational drug repositioning method by performing omics analyses of publicly available expression profile and protein interaction data. Our aim was to find drug candidates for DHF that could prevent dengue virus replication and ameliorate symptoms so that patients with the milder DHF did not progress to the more severe DHF. Our drug repositioning method is not based on clinical knowledge of the drug treatment of dengue symptoms but instead on rational integration of multiple omics data. Combining data produced by different types of high-throughput technologies allowed us to collect information on the molecular components of biological systems. High-throughput technologies are designed to collect large sets of molecular data on specific nucleotide sequences, gene expressions, and protein abundances that are referred to as “omics” data, such as genomics, transcriptomics, proteomics, and interactomics. The large-scale intergradation of experimentally measured data aimed at data-driven science makes it possible to draw a comprehensive view of biological processes and biological networks crossing different molecule layers. Previous multiple omics analyses of this kind have reported some informative results. For example, Chen *et al*.^18^ found various medical risks, including type 2 diabetes, by observing integrated personal omics data (genomic, transcriptomic, proteomic, metabolomic, and autoantibody profiles) over 14 months. Furthermore, in their review, Zhan *et al*.^19^ concluded that an integrated multiple omics approach was a powerful tool for understanding the functional principles and dynamics of total cellular systems for microbial biology.

In this study, we used omics data and bioinformatics techniques to perform a multi-layer integration of three layers of data: transcriptomic, proteomic, and interactomic data. The obtained gene expression pattern was applied to a connectivity map method to select drug candidates for DHF. Using proteomic and interactomic data, we chose drug candidates by finding the host proteins that directly contact dengue virus proteins. The potential drug candidates were narrowed down by integrating the signature molecules, pathways, and drug information from the three layers. The identified drug candidates are expected to induce a suppressed level of gene expression and disrupt the association of host proteins with dengue virus proteins.

## Methods

**Figure 1:**
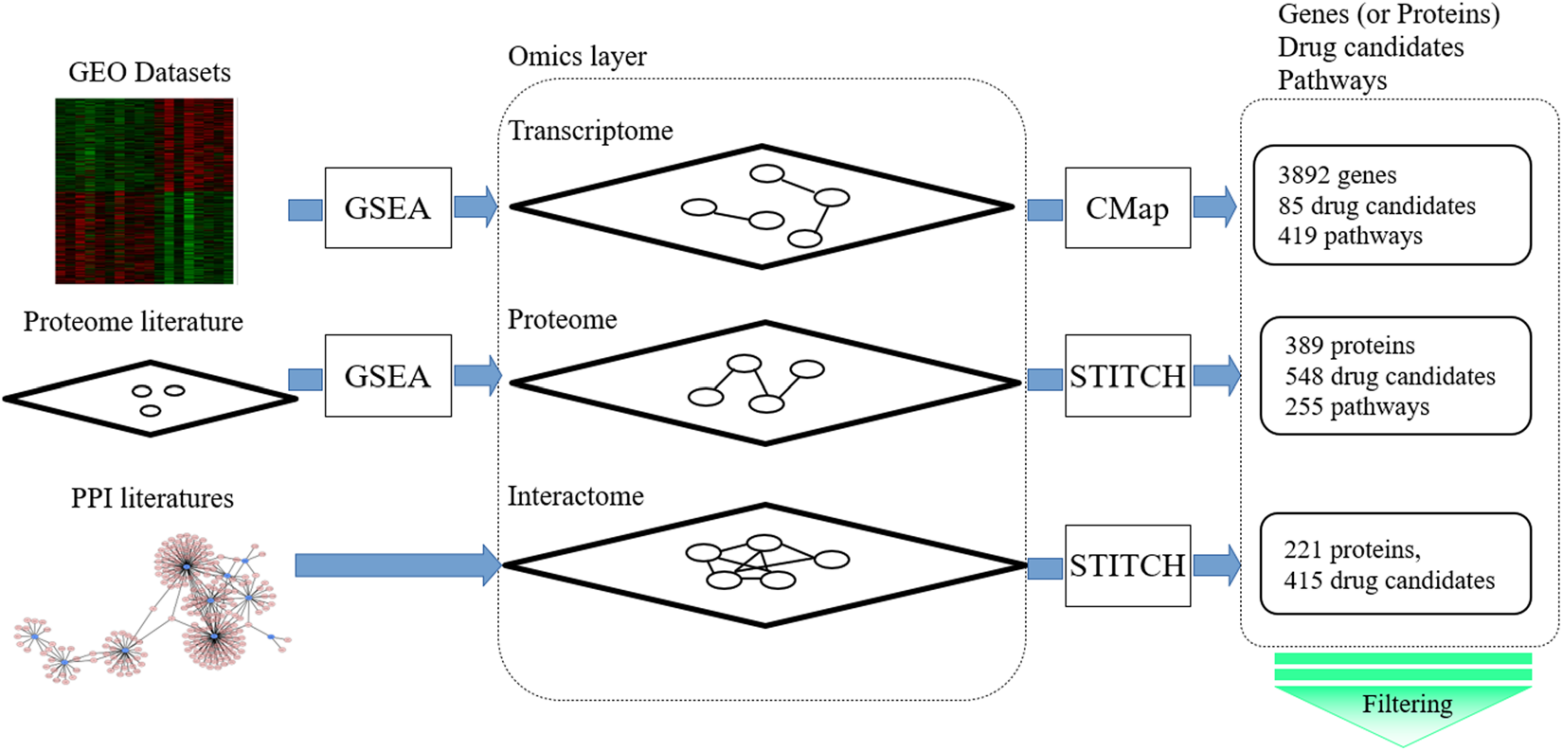
Schematic overview of the integrative analysis of omics data performed in this study to detect potential drug candidates. Firstly, signature genes, signature proteins, and interactions between human proteins and dengue virus proteins were selected from GEO datasets, and by reviewing the proteome literature and human–dengue virus PPI literature, respectively. Secondly, disease-specific pathways for the transcriptome and proteome data were detected by GSEA using the signature genes and proteins identified in the first step. Then, a human–dengue virus PPI network was constructed by integrating the interaction data from the PPI literature. Thirdly, drug candidates were detected in the transcriptomic, proteomic, and interactomic layers by CMap and STITCH searches. Fourthly, a set of drug candidates for DHF was identified by integrating the significant molecules, pathways, and drug candidates obtained in each layer. Finally, we identified common significant molecules and pathways for each omics analysis and layer, and identified the drug candidates targeting the human proteins that interact with dengue virus proteins in a targeted mechanism-based method. These methods are explained in detail in the main text.

A schematic view of our multi-layer analysis method is shown in Fig. 1. Firstly, for the transcriptome analysis, we used gene expression data of patients with DHF from Gene Expression Omnibus (GEO) datasets^20–24^. Then, a connectivity map (CMap)^25,26^ was used to find drug candidates in a signature-based drug repositioning method. Secondly, we searched the literature for proteomic analyses of dengue virus, and selected signature proteins from the relevant studies^27^. We also performed Gene Set Enrichment Analysis (GSEA)^28^ to detect disease-specific pathways for significant differences in gene and protein expressions between dengue patients and normal controls. Thirdly, we searched the literature on protein–protein interactions (PPIs) between human and dengue virus proteins and obtained a network for interactome analysis^29–33^ to identify human proteins that interact with dengue virus proteins. In a target-based method, STITCH^34,35^ was searched to find drugs that could interact with the identified signature proteins from the proteome and human proteins from the interactome.

### Transcriptomic analysis

#### Identification of signature genes between patients with DHF and normal controls

Three microarray gene expression datasets related to purified peripheral blood mononuclear cells from patients with DHF were obtained from the GEO database as follows: GSE18090, from blood samples of eight normal controls (ND; non-disease) and 10 DHF patients^22^; GSE25226, from 10 ND and six DHF patients^23^; and GSE38246, from eight ND and 32 DHF patients^24^. The gene expression data from GSE25226 and GSE38246 were obtained from the blood of children with ND and DHF patients who were less than 15 years old in Nicaragua. In each dataset, we analysed the signature genes with *p* < 0.015 in t-test between the DHF and ND subjects by z-score transformation of the three GEO matrices. The signature genes were determined by integrating the three results.

#### Identification of significant pathways by GSEA

We used the GSEA method^28^ to find statistically significant pathways from the Molecular Signatures Database (MsigDB)^36,37^ and Pathway Ontology (PWO)^38^ for differentially expressed genes and proteins. A hypergeometric test was applied to significant gene groups between DHF and ND to identify the disease-specific pathways in MsigDB and PWO with *p* < 0.05. MsigDB contains gene sets of canonical pathways that were gathered from the BioCarta, KEGG pathway, and Reactome databases. Because these databases have different pathway annotation policies, the gene sets of pathways in each database were different from each other. We used PWO in addition to the canonical pathways in the pathway analysis. For human, PWO is provided by the Rat Genome Database^39^, and its characteristics are presented in a detailed hierarchical structure that has five parent nodes: classic metabolic, regulatory, signalling, drug, and disease pathways. A total of 2,482 pathways have been assigned into the structure based on an extensive survey of the review literature along with searches of existing pathway databases. We performed GSEA with the molecular signatures of the canonical pathway (c2.cp.v5.0.entrez) and PWO (http://bioportal.bioontology.org/ontologies/PW).

#### Identification of drug candidates by CMap

CMap is a collection of genome-wide transcriptional expression data from human cell lines that have been treated with chemical compounds^25^. The simple pattern matching algorithms in CMap enable the discovery of functional connections between drugs, genes, and diseases through the transitory feature of common gene-expression changes. CMap contains the gene expression profiles obtained from five different cell lines treated with 1,309 chemical compounds. For drug discovery for DHF, the probe list of upregulated and downregulated signatures between ND and DHF patients was applied to CMap to identify any inverse drug–disease relationships. The threshold of significance for each drug was set at *p* < 0.1 using the permutated results. In addition, an enrichment analysis of the classes of the obtained drug candidates was performed using the Anatomical Therapeutic Chemical (ATC) classification (https://www.whocc.no/atc/structure_and_principles/) and the Kyoto Encyclopaedia of Genes and Genomes (KEGG) drug groups (DGroup; http://www.genome.jp/kegg/drug/). The ATC classification provided by the World Health Organization has 13 first-level codes that are divided into 94 second-level, 267 third-level, 882 fourth-level, and 4,580 fifth-level codes (that represent individual drugs). KEGG DGroup is part of a new database (currently under development) that describes structurally- and functionally-related groups of drugs, especially from the viewpoint of drug interaction networks. It consists of 503 first-level codes that are divided into 1,252 second-level, 1,685 third-level, 2,753 fourth-level, and 1,685 fifth-level codes. We experimented with different ways of classifying drugs using ATC and KEGG DGroup.

### Proteomic analysis

#### Identification of signature proteins in the cellular response

We searched the literature for proteomic analyses of cells affected by dengue virus on PubMed and Google scholar. We identified relevant studies using the keywords “proteomics analysis” and “proteome”^27,40–45^. Chiu *et al*.^27^ reported a proteomic analysis of human A549 cells in response to infection with dengue virus type 2 using high-throughput mass spectrometry. They identified about 4,000 proteins with significant differences in abundances between dengue virus infected and normal cells. They found that 94.5% and 90% of proteins in the cytoplasmic and nuclear fractions, respectively, showed a >1.5-fold change in abundance following dengue virus type 2 infection. Ray *et al*.^40^ detected 18 proteins by 2D-DIGE in combination with MALDI-TOF/TOF MS in serum cells of dengue fever patients. Using label-free LC-MS, Pando-Robles *et al*.^41^ detected 155 proteins with altered expression in infected Huh-7 cells at 24 h post-infection. Rungruengphol *et al*.^42^ identified 128 proteins from infected LLC-MK2 cells in their study using MALDI-TOF, principal component analysis, and a sub-10 kDa peptidome analysis that used only proteins <10 kDa in size. Fragnoud *et al*.^43^ identified 22 proteins compared proteins purified from plasma pools of dengue fever patients and normal controls. These proteins were analysed by nano-liquid chromatography coupled to ion trap mass spectrometry and identified using data libraries. Huerta *et al*.^44^ found 239 proteins identified in the elution fractions of human plasma subjected to DE-52 anion exchange chromatography on Huh-7.5 cells infected by dengue virus type 2. Caruso *et al*.^45^ studied sub-10 kDa proteins and identified 175 proteins from HepG2 cells. We used all the proteins from cell lines and patient samples identified by these studies in our proteomic analysis. Notably, the proteins reported by Chiu *et al*.^27^ covered all the proteins detected in the other studies.

#### Identification of significant pathways by GSEA

To identify significant pathways, we performed an improved GSEA on the signature proteins of the proteome using the methods described above for the transcriptomic analysis.

#### Identification of drug candidates based on proteomics

STITCH 5.0^34,35^ was used to find interactions between chemical compounds and proteins as an interaction network. STITCH encompasses about 9 million proteins from 2,000 eukaryotic and prokaryotic genomes as well as 430,000 chemical compounds. To find direct interactions, we searched STITCH for significant proteins and obtained a “combined score” that was computed from the four scores of protein-chemical interactions entitled “prediction”, “experimental”, “database” and “textmining” in STTICH^46^. We set the acceptable “combined score” to be >0.7 to ensure a high level of confidence for the interaction. The obtained interaction network accepted only potential drug candidates and extracted the interactions between chemical compounds and significant proteins. Among the 430,000 chemical compounds in STITCH, 2,941 potential drug candidates were identified; 2,230 of them were approved drugs from the FDA and 1,309 were from CMap. There were 598 common drugs between CMap and the FDA.

### Interactomic analysis

#### PPI network between human and dengue virus proteins

Our literature searches found five studies^29–33^ that reported experimental evidence for interactions between human proteins and dengue virus proteins based on high-throughput yeast two-hybrid screening methods. Khadka *et al*.^30^ reported 139 interactions between dengue virus and human proteins, the majority of which were novel. Le Breton *et al*.^31^ focused on the NS3 and NS5 viral proteins, which are the major enzymatic components of the viral replication complex and are essential to the flavivirus life cycle. They reported 186 interactions between dengue virus and human proteins; 171 of the interactions were identified by yeast two-hybrid methods and 16 were from previously published data. Mairiang *et al*.^32^ identified 46 interactions, including six that had been reported previously. Recently, Dey and Mukhopadhyay^33^ reported the development of DenvInt, a database of manually curated experimental data of dengue protein and host protein interactions. We merged the data of published references from DenvInt and used it in our analysis.

#### Drug candidates based on interactomics analyses

To find drug candidates, we searched STITCH 5.0 to identify the compounds that interact with the human proteins involved in human–dengue virus PPIs. The resulting drug candidates are expected to disrupt associations between human proteins and dengue virus proteins.

## Results and Discussion

### Transcriptomic analysis

**Figure 2:**
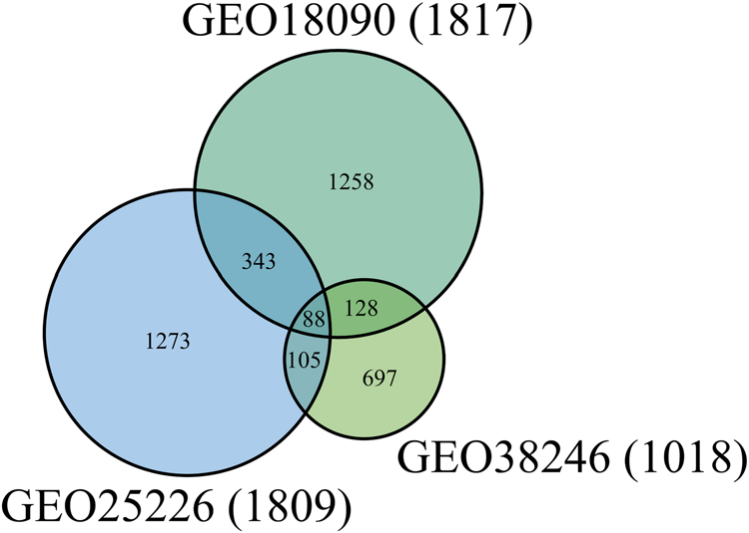
Venn diagram of the signature genes detected in the transcriptomic analysis. The number of signature genes from three GEO gene expression data (GSE18090, GSE25226, GSE38246) are shown in the green, light blue, light green circle, respectively. The numbers of signature genes that overlap between these data are also shown.

We identified 1,817 signature genes with significant differences in expression (*p* < 0.015 in t-test) between the 10 DHF patients and 8 normal controls from the GSE18090 dataset. A heat map of the expression data is shown in Supplementary Fig. S1. We also identified 1,809 and 1,018 signature genes with significant differences in expression between DHF patients and normal controls in Nicaraguan children from the GSE25226 and GSE38246 datasets, respectively. The 3,892 signature genes that we obtained by integrating the results of the three GEO datasets are listed in Supplementary Table S1 and are presented in a Venn diagram in Fig. 2, which shows that 88 signature genes were common among the three datasets. We also analysed the signature genes with expression fold changes >1.5 or <0.666 (Supplementary Fig. S1). Based on the fold changes, 57 signature genes were common among the three datasets. The GSEA of the 3,892 signature genes identified 419 pathways in the canonical pathways in MsigDB and PWO as significant for DHF. Table 1 shows the top 10 pathways; 7 are from the Reactome database, 2 are from KEGG, and 1 is from PWO.

**Table 1:** Top 10 pathways identified by GSEA of the transcriptomic expression data.

Pathway	P-value
REACTOME_CELL_CYCLE	4.78 × 10^−33^
REACTOME_DNA_REPLICATION	1.79 × 10^−28^
REACTOME_CELL_CYCLE_MITOTIC	1.24 × 10^−27^
REACTOME_MITOTIC_M_M_G1_PHASES	1.62 × 10^−24^
PWO_REGULATORY_PATHWAY	4.89 × 10^−20^
REACTOME_S_PHASE	2.52 × 10^−19^
REACTOME_G1_S_TRANSITION	3.40 × 10^−19^
REACTOME_MITOTIC_G1_G1_S_PHASES	6.81 × 10^−19^
REACTOME_SYNTHESIS_OF_DNA	1.58 × 10^−17^
PWO_PROTEIN_DEGRADATION_PATHWAY	1.88 × 10^−17^

The probe list of the top 300 upregulated and bottom 300 downregulated signature genes from GSE18090 that were compatible with the HG-U133A platform of CMap, was used to query the CMap system. Using the CMap permutated results, 85 compounds with statistical significance (*p* < 0.1) were identified, and 40 of them were assigned with ATC codes and KEGG DGroups. We classified the selected compounds based on drug use, and analysed the distribution of both the ATC codes and KEGG DGroups for these compounds (Supplementary Fig. S3). At the first level of the anatomical main group in the hierarchy of ATC classification, 29 of the 40 compounds were assigned to the top six groups, namely “C” (cardiovascular system; 11 compounds), “S” (sensory organs; 6 compounds), “N” (Nervous system; 5 compounds), “D” (dermatologicals; 5 compounds), “J” (anti-infectives for systemic use; 5 compounds), and “A” (alimentary tract and metabolism; 5 compounds). In KEGG DGroups, 30 of the 40 compounds were assigned to the top five groups, namely “Antibacterial” (16 compounds), “Cyp substrate” (12 compounds), “Cardiovascular agent” (11 compounds), “Neuropsychiatric agent” (9 compounds), and “Other” (5 compounds).

### Proteomic analysis

We used the 389 signature proteins reported by Chiu *et al*.^27^ in the proteomic analysis (Supplementary Table S2). The GSEA was applied to the significant proteins in the same manner as in the transcriptomic analysis. A total of 255 statistically significant (*p* < 0.05) pathways were identified, and the top 10 pathways are shown in Table 2. Three of the drug pathways identified in PWO, the etoposide, sorafenib, and irinotechan pathways, are for drugs that are known antineoplastic agents. The drug pathways were found to be statistically significant (*p* < 0.05) by a chi-squared test at the first level of the hierarchy in pathway classification of PWO. Previously, Chiu *et al*.^27^ analysed the statistical significance of these pathways based on Gene Ontology and found that the protein degradation pathway was statistically significant. This pathway was also statistically significant (*p* = 0.0028) in our analysis.

**Table 2:** Top 10 pathways identified by GSEA of the proteomics data.

Pathway	P-value
REACTOME_ANTIVIRAL_MECHANISM_BY_IFN_STIMULATED_GENES	6.94 × 10^−8^
PWO_ETOPOSIDE_DRUG_PATHWAY	3.34 × 10^−7^
PID_FOXM1_PATHWAY	4.66 × 10^−7^
REACTOME_KINESINS	1.37 × 10^−6^
PWO_IRINOTECAN_DRUG_PATHWAY	1.37 × 10^−6^
PWO_SORAFENIB_DRUG_PATHWAY	1.51 × 10^−6^
PID_PLK1_PATHWAY	1.65 × 10^−6^
PWO_AVITAMINOSIS_DISEASE_PATHWAY	2.88 × 10^−6^
PID_MYC_ACTIV_PATHWAY	3.92 × 10^−6^
REACTOME_CELL_CYCLE	6.46 × 10^−6^

By searching STITCH 5.0 for drug candidates based on the proteomic data, we found 548 drug candidates that interacted with the 389 significant proteins. Of these, 412 compounds were assigned ATC codes and KEGG DGroups (KEGGID). The top five ATC groups were “C” (cardiovascular system), “L” (antineoplastic and immunomodulating agents), “A” (alimentary tract and metabolism), “G” (genitourinary system and sex hormones), and “S” (sensory organs), and contained 93, 90, 85, 82, and 81 compounds, respectively. The top five KEGG DGroups were “Cyp substrate”, “Other”, “Antibacterial”, “Cardiovascular agent”, and “Neuropsychiatric agent”, and contained 115, 92, 90, 79, and 63 drug candidates, respectively. The distribution of the compounds in ATC and KEGG DGroups was different (correlation coefficients −0.173 and −0.297, respectively) between the drug candidates obtained from the transcriptome analysis and those obtained from the proteome analysis. This is because the drug candidates from the transcriptomic analysis were identified based on the effect of the drug–disease relationship on the gene expression profiles in patient data (DHF vs ND), whereas the candidates from the proteomic analysis were obtained based on the relationship between chemical compounds and proteins in the cellular response.

### Interactomic analysis

**Figure 3:**
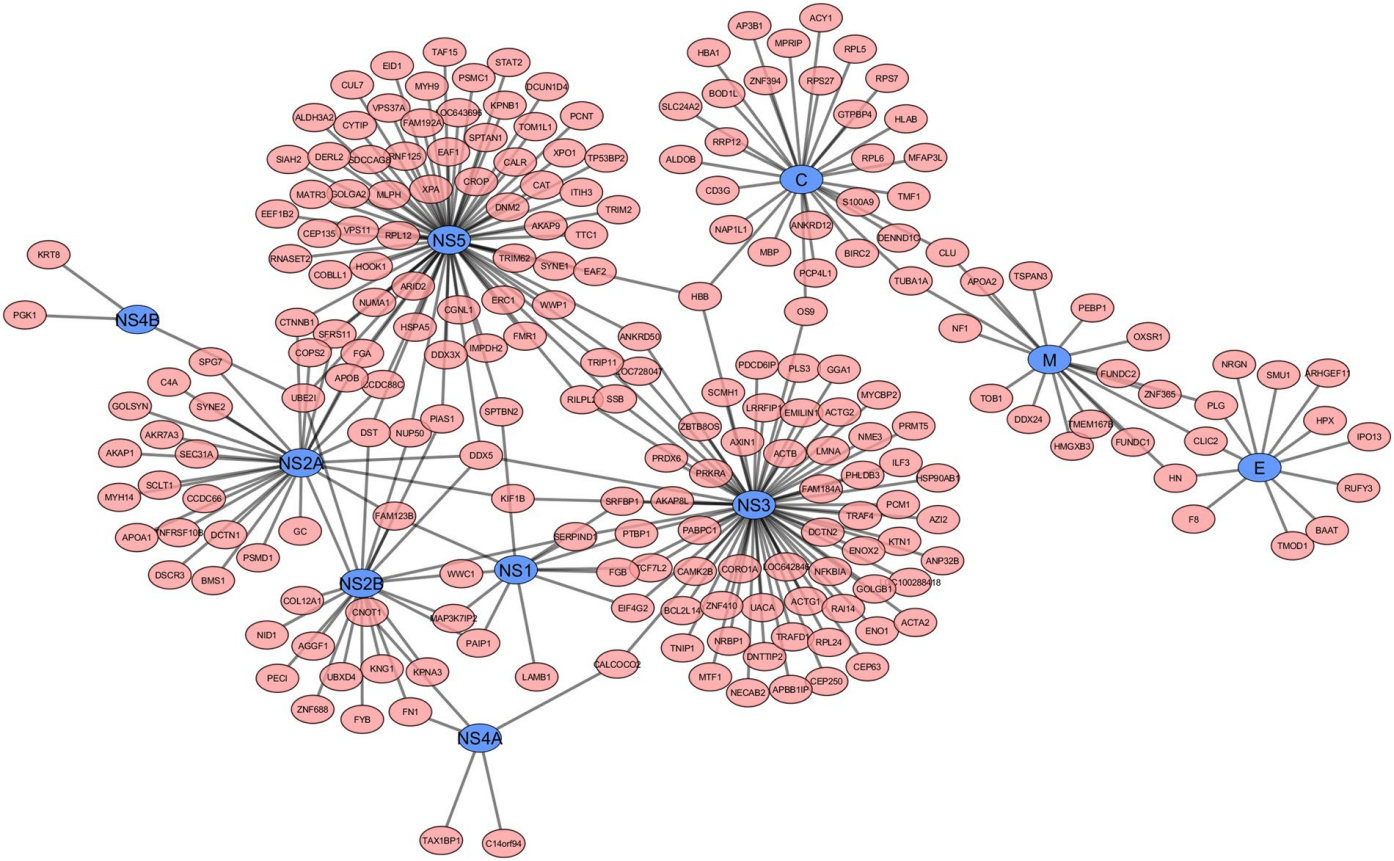
Human protein and dengue virus protein interaction network. Blue nodes represent the dengue viral proteins and are labelled with the corresponding gene names. Pink nodes represent the human proteins and are labelled with the corresponding UniProt ID. The black edges show the interactions between human proteins and dengue viral proteins as determined by our interactomic analysis of the experimental data.

We searched the literature for reported PPIs between human and dengue virus and found several relevant publications^29–33^. The proteins reported in these dengue virus–human PPI studies included all 10 dengue virus proteins (capsid protein C, membrane protein M, envelope protein E, nonstructural protein (NS)1, NS2a, NS2b, NS3, NS4a, NS4b, and NS5). Folly *et al*.^29^ reported 36 human–dengue virus protein interactions that included the dengue virus C, M, and E proteins. Khadka *et al*.^30^ reported 139 human–dengue virus protein interactions that included the dengue virus M, NS1, NS2a, NS2b, NS3, NS4a, NS4b, and NS5 proteins. Le Breton *et al*.^31^ reported 186 human–dengue virus protein interactions that included the dengue virus NS3 and NS5 proteins. Mairiang *et al*.^32^ reported 46 human–dengue virus protein interactions that included the dengue virus C, NS3, and NS5 proteins^29–32^. We integrated the PPI data from these studies and obtained a total of 268 PPIs (Supplementary Table S3), which involved all 10 dengue virus proteins and 221 human proteins. The human–viral PPI network incorporating these identified PPIs is shown in Fig. 3. The top five dengue virus proteins in terms of the number of interactions with human proteins were NS5, NS3, C, NS2A, and NS2B, which displayed 72, 68, 29, 28, and 20 interactions, respectively. Eight of the 10 virus proteins interacted with more than 10 human proteins. NS4B had the lowest number of interactions with only three. Human protein DDX5 interacted with four virus proteins, the highest number of interactions, followed by 5 and 32 human proteins that interacted with three and two virus proteins, respectively. By contrast, 183 human proteins interacted with only one virus protein each.

In searching for drug candidates from the interactomic data, we found 415 drug candidates in STITCH that interacted with the 221 human proteins identified in the human–viral PPIs. Of these 415 compounds, 315 were assigned with ATC codes and identified with KEGG DGroups. We analysed the distribution of these ATC codes and KEGG DGroups in the same manner as in the proteome analysis described above. For the ATC codes, the top five anatomical main groups were “C” (cardiovascular system), “A” (alimentary tract and metabolism), “G” (genitourinary system and sex hormones), “L” (antineoplastic and immunomodulating agents), and “S” (sensory organs), and contained 77, 75, 69, 61, and 59 compounds, respectively. For the KEGG DGroups, the top five groups in the first level were “Cyp substrate”, “Cardiovascular agent”, “Other”, “Antibacterial”, and “Neuropsychiatric agent”, and contained 85, 72, 69, 46, and 39 compounds, respectively. The distributions of compounds in ATC and KEGG DGroups between the proteomics analysis and the interactomic analysis were similar (correlation coefficients 0.888 and 0.832, respectively). The high number of overlapped proteins between the proteome and interactome analyses clearly is responsible for the similarity in the distribution of the compounds obtained from STITCH.

### Multiple omics analysis

**Figure 4:**
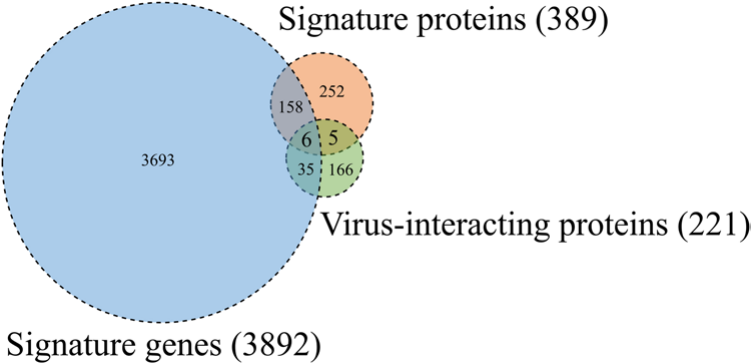
Venn diagram of signature genes, signature proteins, and human proteins that interact with dengue virus proteins. The number of signature genes obtained from the gene expression data with significant differences between DHF patients and normal controls is shown in the blue circle. The number of signature proteins obtained from the protein expression data is shown in the orange circle. The number of human proteins that interact with dengue virus proteins in human–virus PPIs is shown in the green circle. The numbers of gene products and proteins that overlap between the groups are also shown.

We conducted a multiple-step comparison of the 3,892 significant genes identified by the transcriptomic analysis, 389 proteins identified by the proteomic analysis, and 221 human proteins identified by the human–dengue virus PPIs as shown in Fig. 4. The genes and proteins are listed in Supplementary Table S4. We found that 41 proteins overlapped between the signature gene products and the human proteins from the human–virus PPIs, and 11 proteins overlapped between the signature proteins and the human proteins from the human–virus PPI network, namely ACTG1, CALR, CLU, ERC1, HSPA5, KTN1, NUP50, PABPC1, PAIP1, RRP12, and SYNE2. Notably, six of these proteins (CALR, ERC1, HSPA5, KTN1, NUP50, and SYNE2) are likely to play important roles in the infectious mechanisms of DHF. *HSPA5*, also known as *GRP78*, encodes a 78-kDa glucose-regulated protein, which is the HSP70 molecular chaperone in the endoplasmic reticulum. Previous transcriptome and proteome analyses have shown that *HSPA5* was upregulated and a 78-kDa glucose-regulated protein was enriched in dengue virus-infected cells^47–49^. It has been suggested that the 78-kDa glucose-regulated protein may be a component of the dengue virus receptor complex that supports dengue virus entry or facilitates viral protein production^48,50^. CALR and ERC1 are two of six significant proteins in replication of a dengue virus replicon. *CALR* encodes calreticulin, which colocalized with viral dsRNA and with the viral NS3 and NS5 proteins in dengue virus-infected cells, consistent with a direct role for calreticulin in dengue virus replication^30^. *NUP50* encodes nuclear pore protein Nup50, which is a component of the nuclear pore complex. The nuclear pore complex is involved in transporting the dengue virus genome and has been suggested as a therapeutic target for many virus infections^51^. *KTN1* encodes kinectin, which is an endoplasmic reticulum protein that extends the endoplasmic reticulum along microtubules. The endoplasmic reticulum is known to be a central organelle in dengue virus replication. Our transcriptomics, proteomics, and interactomics analyses identified these four proteins as significant proteins in dengue infection.

**Figure 5:**
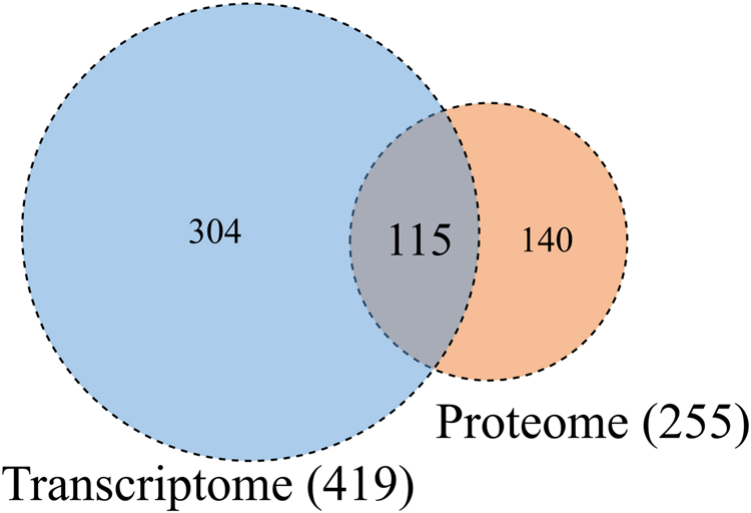
Venn diagram of the significant pathways in the transcriptomic and proteomic analyses. The numbers of significant pathways identified by GSEAs of the transcriptome and proteome are shown in the blue and orange circles, respectively. The number of pathways that overlap between the two analyses is also shown.

**Figure 6:**
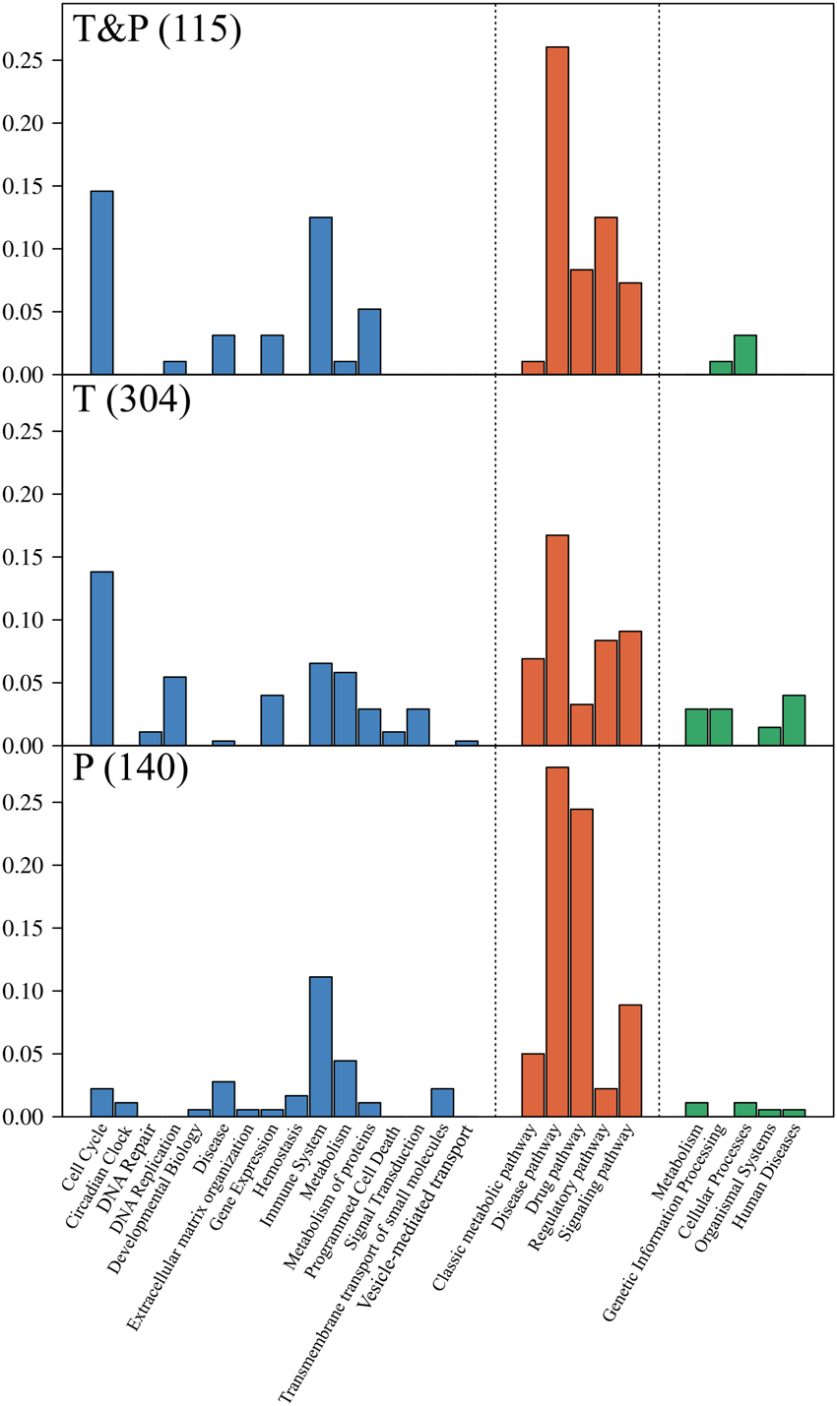
Histograms showing the normalized distribution probability of pathways identified by transcriptomics alone, proteomics alone, and the combination of transcriptomics and proteomics. The bars show the proportion of each group among the total number of pathway types identified by Reactome (blue), PWO (orange), or KEGG (green). The top panel shows the distribution of the 115 common pathways identified by both transcriptomics and proteomics GSEAs (T&P). The middle panel shows the distribution of the 304 pathways identified only by the transcriptomics GSEA (T). The bottom panel shows the distribution of the 140 pathways identified only by the proteomics GSEA (P). The sum of all the groups in each section is 1.0 for all three panels.

Figure 5 shows a comparison of the expression profiles for the pathways identified by GSEA of the transcriptomic and proteomic data, which revealed 115 pathways as common between the two analyses (Supplementary Table S5). A total of 559 detected pathways were categorized into Reactome, PWO, and KEGG pathways for hierarchical analysis (Fig. 6). We examined the distribution of assigned pathways in the first hierarchical level to study the coarse-grained functions for a large set of pathways. Based on the distributions shown in Fig. 6, we assigned the pathways that either existed only among the common pathways or that increased the ratio drastically in these pathways. Specifically, among the 115 common pathways, “Metabolism of proteins,” which is a parent of the “Unfolded protein response” in Reactome, “Regulatory pathway,” which is a parent of the “Protein degradation pathway” in PWO, and “Cellular Processes and Human Diseases” in KEGG, were obtained from the transcriptome and proteome analyses and may play central roles in the infectious mechanism of dengue virus. Furthermore, the top three common pathways in the combined transcriptomics and proteomics analysis (Fig. 6, top panel) are “Drug pathway”, “Regulatory pathway”, and “Metabolism”. The “Drug pathway” in PWO is a pharmacokinetics and pharmacodynamics pathway that is elicited by the administration of specific drugs.

**Figure 7:**
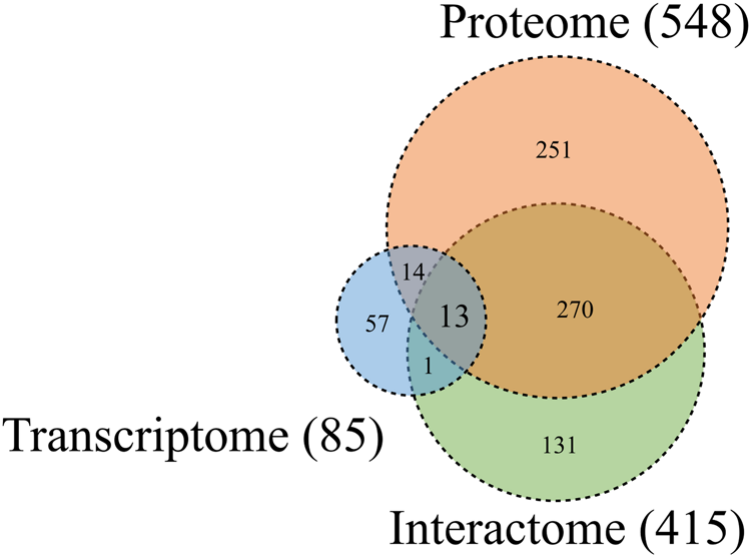
Venn diagram for the drug candidates identified by the transcriptomic, proteomic, and interactomic analyses. The numbers of drug candidates identified by the transcriptomic, proteomic, and interactomic analyses are shown in the blue, orange, and green circles, respectively. The numbers of drug candidates that overlap between each analysis type are also shown.

Figure 7 shows the drug candidates selected using CMap with gene expression profiles and interactomic relationships between signature proteins and chemical compounds. In DHF vs ND, 85 drug candidates were found by CMap. To identify drug candidates in the proteomic analysis, we searched STITCH 5.0 and identified 548 drug candidates that interacted with the 389 significant proteins selected by the protein expression data. For the interactome, we applied the same method to the 221 human proteins found to interact with dengue virus proteins in the human–dengue virus PPIs and obtained 415 drug candidates. Finally, we detected 13 drug candidates that overlapped over the analyses of the three layers (Fig. 7).

**Figure 8:**
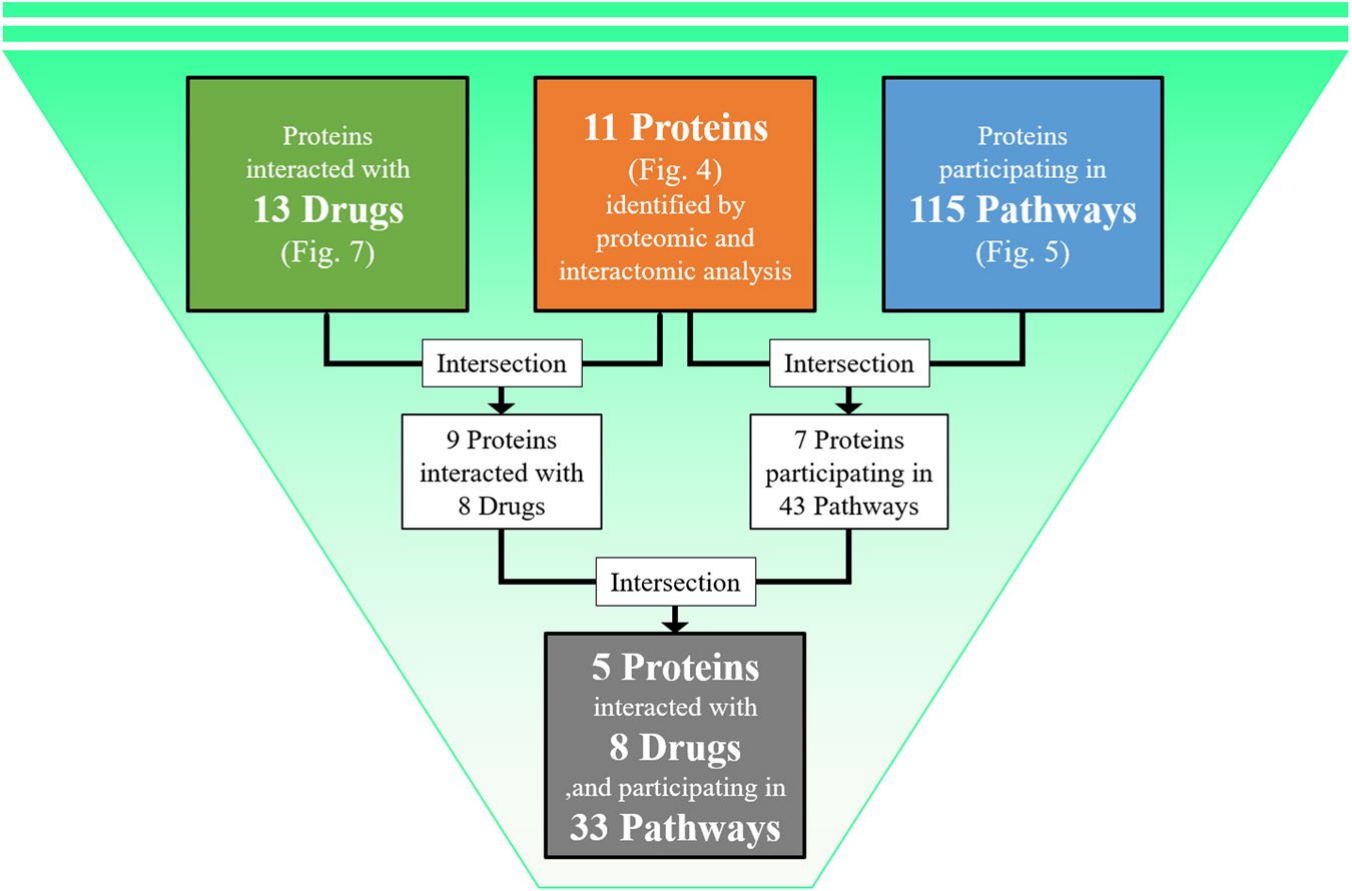
Schematic diagram of the filtering method used to narrow down the drug candidates. 13 drugs were identified as the intersection of the drug candidates by transcriptomic, proteomic and interactomic analyses (Fig. 7). 11 proteins were identified as the intersection between signature proteins of proteomic analysis and human proteins in human-viral PPI (Fig. 4). 115 pathways were identified as the intersection between significant pathways determined by GSEA of transcriptomic analysis and that of proteomic analysis (Fig. 5). Firstly, nine proteins were selected as the intersection between proteins interacted with 13 drugs and 11 signature proteins. These nine proteins were interacted with eight drugs out of 13 drugs. Secondly, seven proteins were selected as the intersection between proteins participating in 115 pathways and 11 signature proteins. These seven proteins were mapped in 43 pathways out of 115 pathways. Finally, five proteins were selected as the intersection between nine proteins interacted with 13 drugs and seven proteins participating in 43 pathways. These five proteins were interacted with eight drugs, and were participating in 33 pathways out of 43 pathways. Then, eight drugs were identified as candidates for use in future drug repositioning.

**Figure 9:**
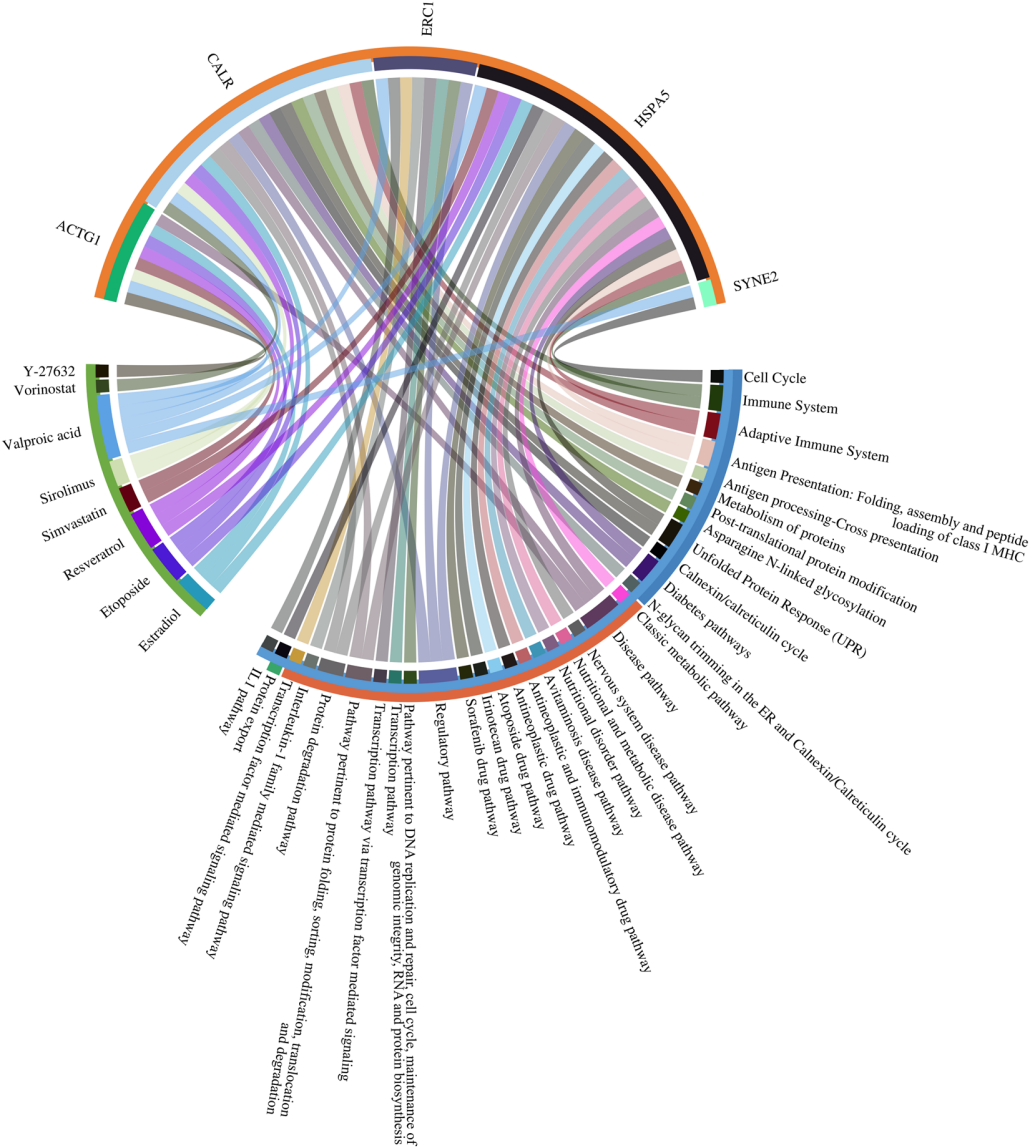
Chord diagram of the likely relationships among drug candidates, proteins, and pathways identified by the multiple omics analyses. Based on the drug repositioning method, eight drugs, five proteins, and 33 pathways were selected as potential candidates for use in the development of treatments for DHF. The drug candidates, proteins, and pathways are shown in green, orange, and cyan, respectively. Connections show the interactions among the drug candidates, proteins, and pathways. The Reactome, PWO, and KEGG pathways are shown in blue, orange, and green, respectively.

A diagram of the process used to filter and narrow down the drug candidates based on the common proteins (union of proteins in Fig. 4), the common pathways (union of pathways in Fig. 5), and the common drugs (union of drugs in Fig. 7) of three layers is shown in Fig. 8. This process yielded 11 target proteins, 115 target pathways, and 13 drug candidates. Based on the interactions between the drug candidates and the target proteins, we found eight drug candidates and nine proteins. When we focused on the proteome and transcriptome analyses, we found that seven of the 11 target proteins could be mapped in 43 of the 115 target pathways. By combining the above findings, we narrowed down the number of drug candidates that targeted the human proteins in human–dengue virus PPIs and the signature proteins in the proteomic analysis mapped on 33 significant pathways to only eight. We found that five proteins (ACTG1, CALR, ERC1, HSPA5, SYNE2) out of the seven could be mapped to these 33 pathways. These five proteins interacted with one viral protein each. For example, ACTG1 interacted with NS3; CALR, ERC1, and HSPA5 interacted with NS5; and SYNE2 interacted with NS2A, as shown in Fig. 3. The likely relationships among drug candidates, proteins, and pathways are shown in Fig. 9. The eight identified drugs are likely to be effective for DHF and may be suitable for drug repositioning for this purpose. We described the list of the eight drug candidates in Table 3. Those structures and clustering analysis are shown in Supplementary Fig. S6(a,b).

**Table 3:** The eight drug candidates identified by multiple omics analysis in this study.

Name	Activity	Disease	ATC	KEGG DGroup
Estradiol	Menstruation disorder agent, Estrogen receptor agonist	Postmenopausal osteoporosis	Genito urinary system and sex hormones (G)	Other, Cyp substrate
Etoposide	Antineoplastic, Topoisomerase II inhibitor	Testicular tumors, Small cell lung cancer	Antineoplastic and immunomodulating agents (L)	Other, Cyp substrate
Resveratrol	Antiinflammatory agents	Herpes labialis infections		
Simvastatin	Antihyperlipidemic, HMG-CoA reductase inhibitor	Hyperlipidemia, Heterozygous familial hypercholesterolemia	Cardiovascular system (C)	Cardiovascular agent, Cyp substrate
Sirolimus	Antineoplastic, Immunosuppressant, mTOR inhibitor	Lymphangioleiomyomatosis	Antineoplastic and immunomodulating agents (L), Sensory organs (S)	Antineoplastic, Transporter substrate
Valproic acid	Anticonvulsant	Seizure disorders, Migraine headache.	Nervous system (N)	Neuropsychiatric agent, Other, Cyp substrate
Vorinostat	Antineoplastic, Histone deacetylase inhibitor	Cutaneous T-cell lymphoma	Antineoplastic and immunomodulating agents (L)	
Y-27632	ROCK family kinase inhibitor, hiPS/ES Cell death inhibitor			

Five drug candidates, valparoic acid, sirolimus, resveratrol, vorinostat, and Y-27632, out of the eight identified in this study have already been reported as effective in inhibiting infections with other flaviviruses. Valparoic acid inhibited contact with the E protein of other flaviviruses, so it is expected to be similarly effective against dengue virus^52^. Sirolimus inhibited viral growth and viral protein expression in flaviviruses^53,54^. Resveratrol exerted a negative effect on dengue virus replication^55^. Vorinostat was shown to have a potential synergistic effect for the treatment of West Nile virus encephalitis^56^. Y-27632, a Rho-associated coiled-coil forming kinase (ROCK) inhibitor, was found to block dengue virus type 2 infection^57^. The likely relationships and connections among the identified drug candidates, proteins, and pathways based on their categorization by Reactome, PWO, and KEGG are shown in Fig. 9. The order of connections from the eight drugs to the five proteins is valparoic acid, resveratrol, estradiol, and diethylstilbestrol, respectively. The remaining drug candidates have an order of one or two in the connections. Valproic acid is the drug most expected to inhibit dengue infection, and it has been previously demonstrated to be an inhibitor of other flaviviruses. The seven drugs except vorinostat interact with ACTC1 which is the highest number of the connections to the drugs among those five. ACTC1 has a role of reorientation cytoskeleton and interacting with vimentin. It is known that rearrangement of the cytoskeleton in host cells is closely involved in the virus life cycle, starting from virus entry to egress. Vimentin is the major component of mesenchymal cells. The vimentin rearrangement induced by dengue infection can be blocked by the inhibitor drugs. Figure 9 is broken down in Supplementary Figs S4 and S5 to show the relationships based on Reactome and PWO separately. HSPA5 has the highest number of connections to the represented pathways. It is known that the unfolded protein response is a pro-survival cellular reaction induced in response to dengue virus-mediated endoplasmic reticulum stress^58,59^. We found that these pathways were related directly to the unfolded protein response. These pathways are included in the Reactome as “activation of chaperones by ATF6-alpha” and “activation of chaperone genes by ATF6-alpha” as well as in PWO as “pathway pertinent to protein folding, sorting, modification, translocation and degradation” and “protein degradation pathway”. HSPA5 and CALR are always involved in these pathways. They interact with chaperone proteins and regulate their functions in protein folding and protein degradation. The seven drugs except Y-27632 interact with the proteins annotated to the unfolded protein response. A potential anti-dengue virus agent that targets HSPA5 has been developed. HSPA5 localized to the cell surface and associated with dengue virus receptor complexes and was shown to block entry of the dengue virus by disrupting the association with the dengue virus receptor complex^60^. The drug candidates identified in our study are expected to induce a suppressed level of gene expression and disrupt the association of host proteins with dengue virus proteins.

Our computational approach using multiple omics data for drug repositioning successfully detected the five drugs out of eight that have already been used to treat infectious diseases caused by flaviviruses. The five reported drugs are all non-anticancer agents. Excluding the anti-cancer agents in Table 3, we found the two drugs, estradiol and simvastatin which have not been reported previously for the treatment of flaviviruses. The seven drug candidates are considered to be strong candidates of drug repositioning to prevent dengue virus replication or ameliorate symptoms.

## Conclusions

We computationally analysed omics data to find effective drug candidates for drug repositioning in DHF by integrating transcriptomic, proteomic, and interactomic experimental data. In each layer, we obtained signature molecules from the omics data, as well as pathways and drug candidates. Some significant molecules that were identified as important for dengue infection confirmed results reported previously. The pathways in dengue infection obtained by modified GSEAs of transcriptomics and proteomics data showed significance in the “unfolded protein response”. The drug candidates were selected using a drug repositioning method that applied gene expression profiles for an inverse drug–disease relationship as well as the interactomic relationships between signature proteins and chemical compounds. Finally, by combining information on relevant pathways and FDA-approved drugs, we narrowed down the identified drugs to eight drug candidates for drug repositioning. Five of these drugs, valparoic acid, sirolimus, resveratrol, vorinostat, and Y-27632, have been reported previously as effective treatments for flavivirus-induced diseases. We consider the present computational approach can be used effectively to identify novel drug candidates that have a strong relationship in multiple omics. However, future studies are needed for validation of the drug effect in dengue-infected cells/patients.

As part of the continued study of this and related procedures, we are exploring some other mosquito-borne diseases, such as zika, chikungunya, and West Nile fever. Improvements in high-throughput experimental technology and the accumulation of data will make it possible to use other omics data such as metabolomics and post-translational modifications, phospholomics, and methylomics. The integration of multiple omics analyses may help reveal the relationships among mosquito-borne diseases, signature molecules, pathways, and drugs. This work will assist with efforts using open scientific data to identify drugs suitable for drug repositioning/repurposing to treat neglected tropical diseases.

## Electronic supplementary material

Supplementary Information

Supplementary Dataset 1

Supplementary Dataset 2

Supplementary Dataset 3

Supplementary Dataset 4
