# Supplementary material for: Drug repositioning for dengue haemorrhagic fever by integrating multiple omics analyses
Source: Sci Rep. 2019 Jan 24;9:523. doi: 10.1038/s41598-018-36636-1 (PMC6346040; doi:10.1038/s41598-018-36636-1)
Supplement: Supplementary file 1 — Supplementary Information [file 41598_2018_36636_MOESM1_ESM.pdf]

Supplementary Information

Drug repositioning for dengue haemorrhagic fever by integrating multiple omics analyses

Takayuki Amemiya[1,*], M. Michael Gromiha[1,2], Katsuhisa Horimoto[1], Kazuhiko Fukui[1,*]

[1]*Molecular Profiling Research Center for Drug Discovery (molprof), National Institute of Advanced Industrial Science and Technology (AIST), Tokyo 135-0064, Japan*

[2]*Department of Biotechnology, Indian Institute of Technology Madras, Tamil Nadu 600 036, India*

e-mail: takayuki-amemiya@aist.go.jp, k-fukui@aist.go.jp

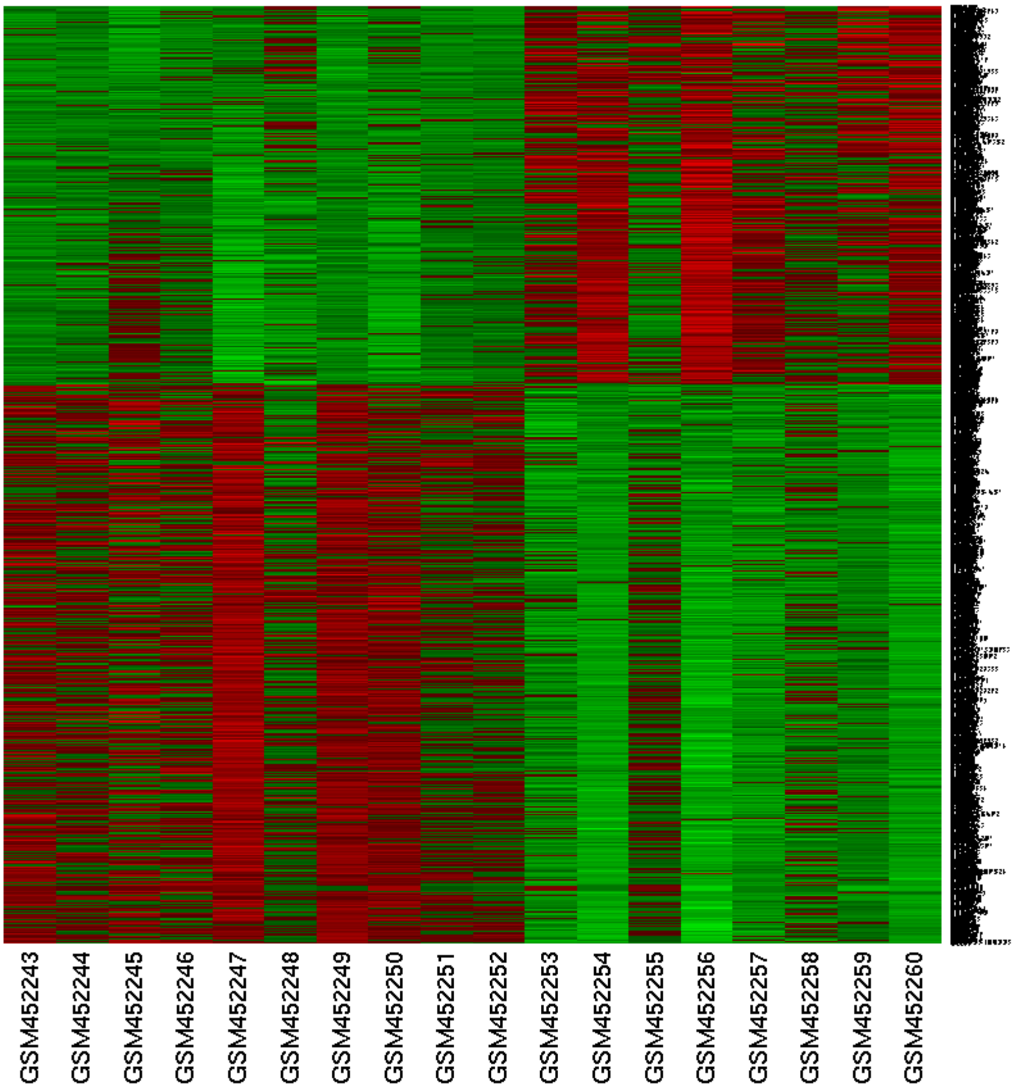

Supplementary Fig. S1. Heat map of signature genes with significant differences in expression between DHF patients and normal controls in the GSE18090 dataset. The heat-map shows gene expression differences between in 10 DHF patients and 8 normal control patients. Red, white, and green indicate upregulated, unchanged, and downregulated expression, respectively, in DHF patients compared with the controls. The genes corresponding to each row of the heat map are listed on the right side of the map.

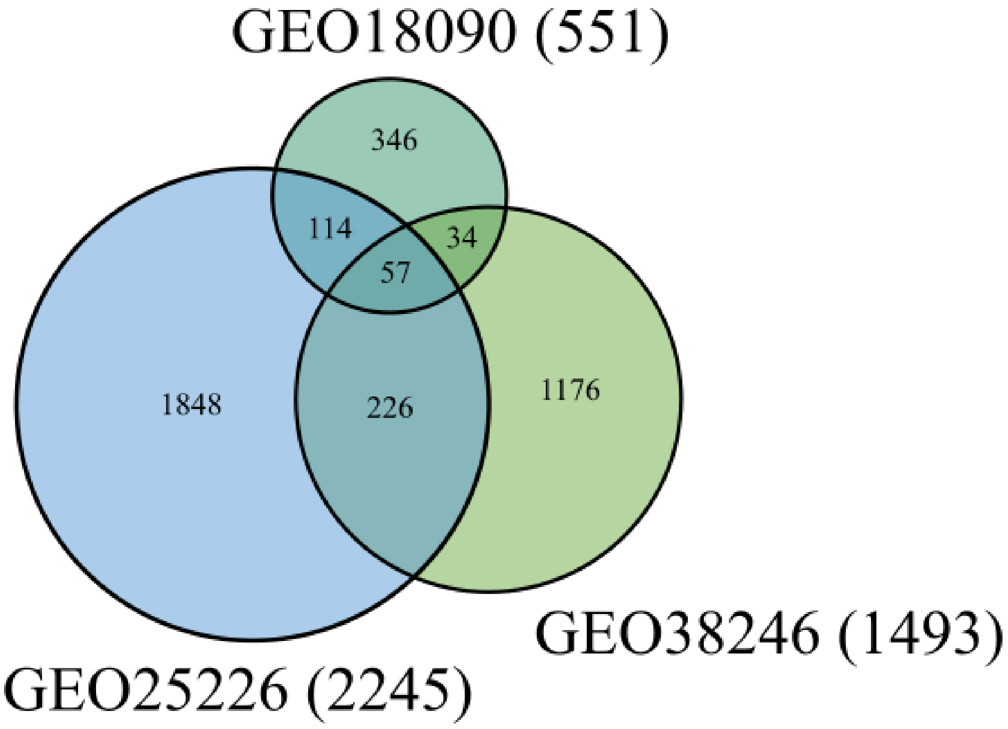

Supplementary Fig. S2. Venn diagram of the signature genes detected in the fold change > 1.5 or < 0.5. The number of signature genes from three GEO gene expression data (GSE18090, GSE25226, GSE38246) are shown in the green, light blue, light green circle, respectively. The numbers of signature genes that overlap between these data are also shown.

(a) ATC code

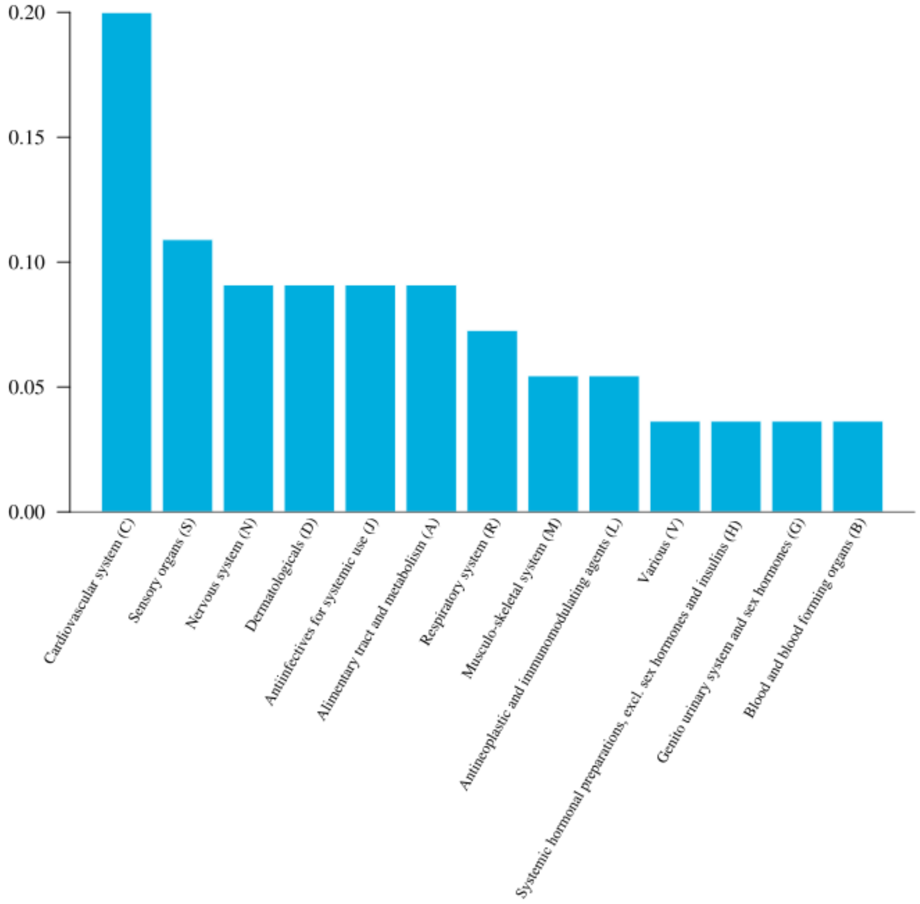

(b) KEGG DGroup

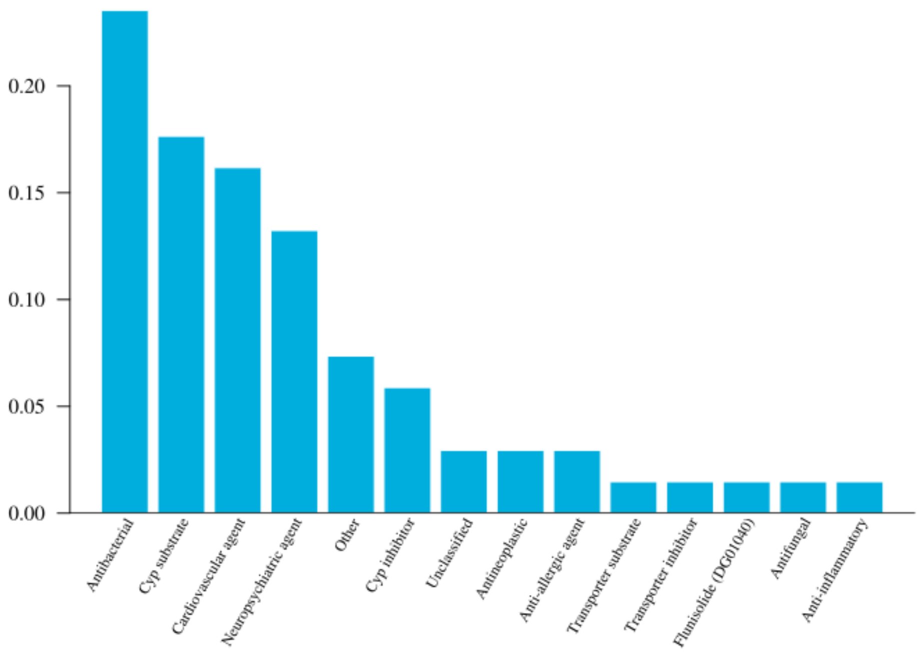

Supplementary Fig. S3. Distribution of ATC code and KEGG DGroup of 105 drug candidates with statistical significance identified by CMap. The bars show the proportion of drug candidates in first level of ATC codes (a) and KEGG DGroups (b). The total proportion is 1.0 for both the ATC codes and KEGG DGroups.

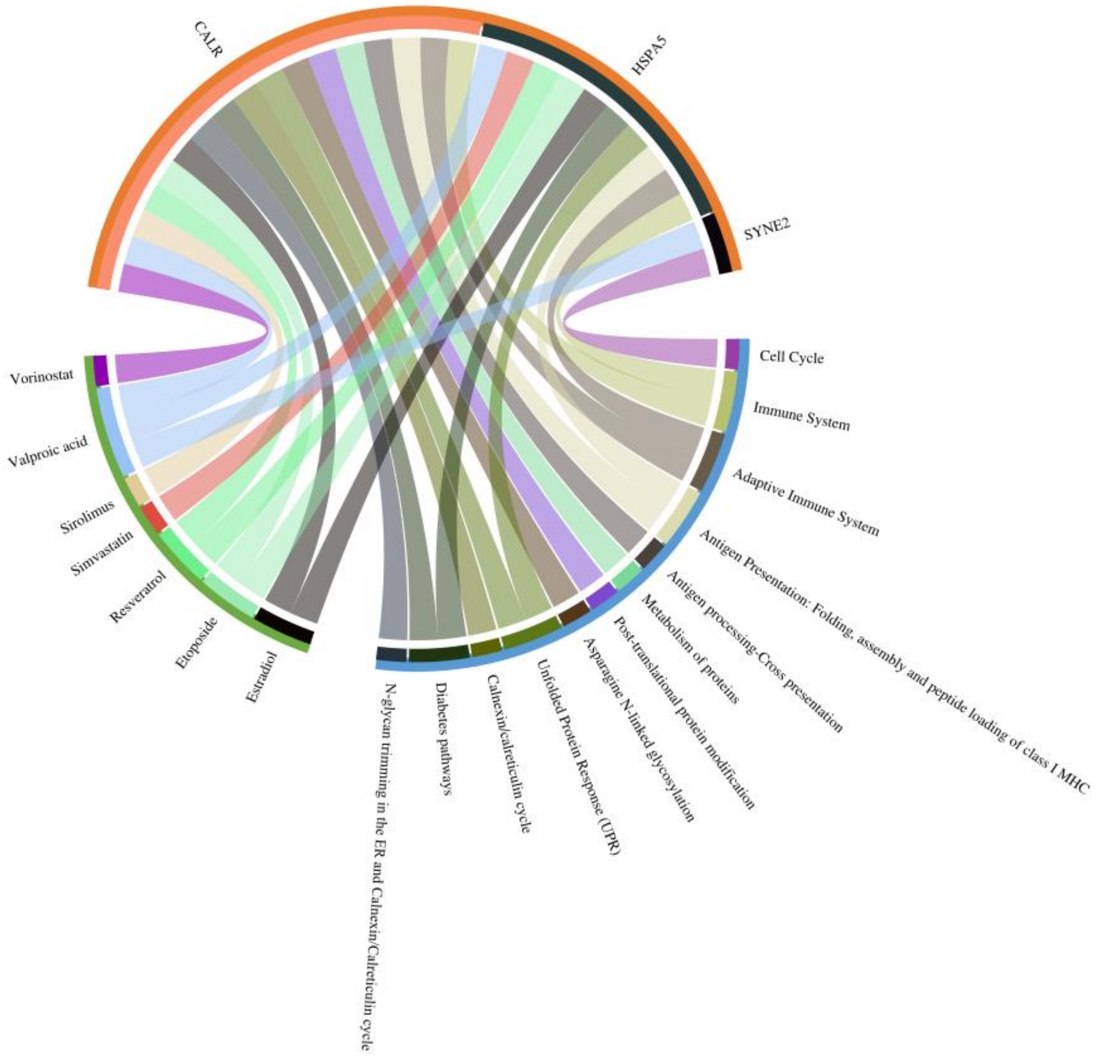

Supplementary Fig. S4. Chord diagram of the likely relationships among drug candidates, proteins, and pathways based on Reactome data. The 12 pathways that were identified in the Reactome database are shown. These pathways were extracted from the diagram in Fig, 9.

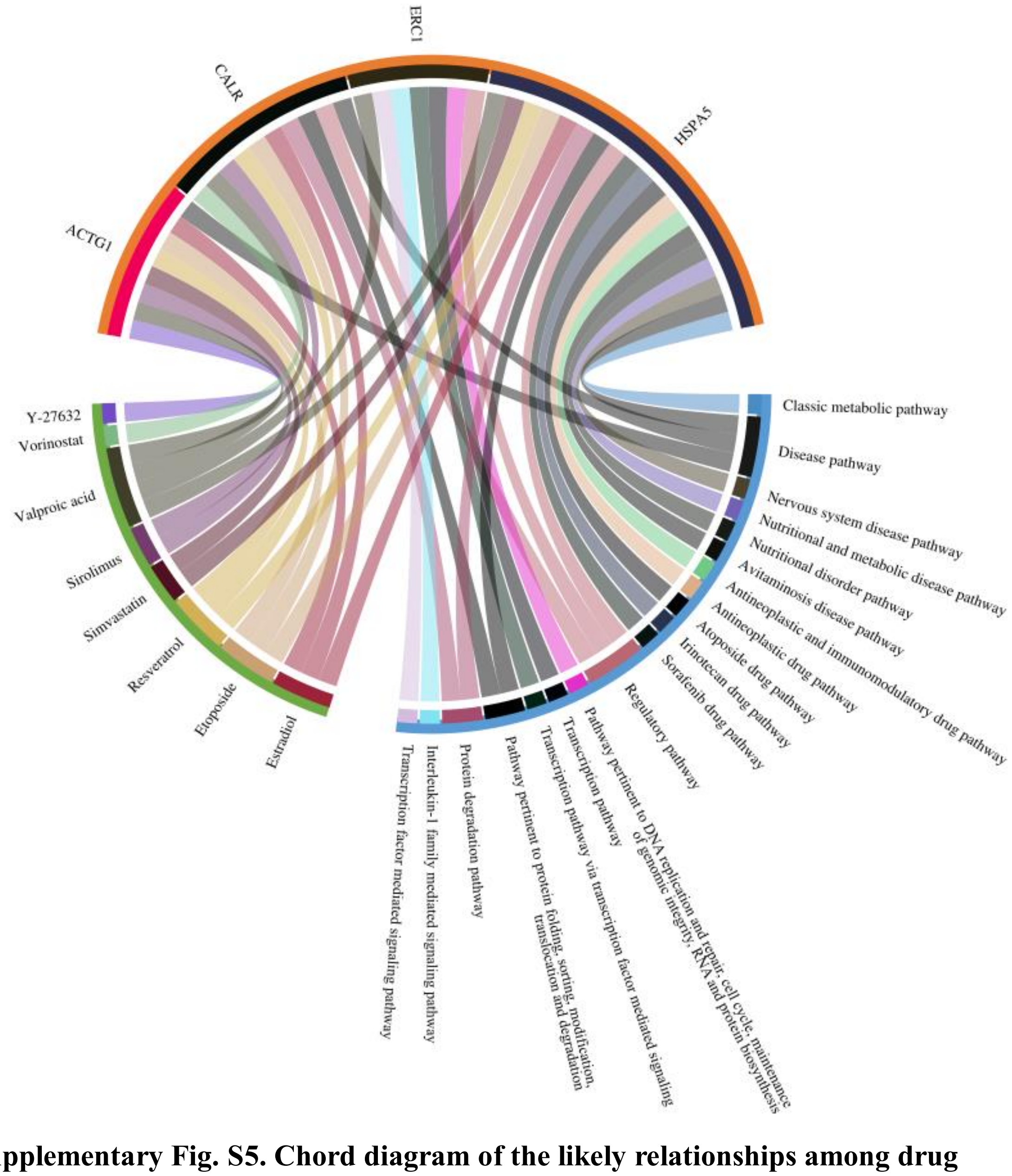

Supplementary Fig. S5. Chord diagram of the likely relationships among drug candidates, proteins, and pathways based on PWO data. The 19 pathways that were identified in PWO are shown. These pathways were extracted from the diagram in Fig. 9.

(a)

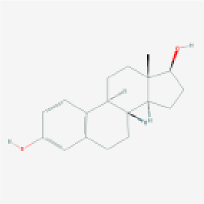

Estradiol

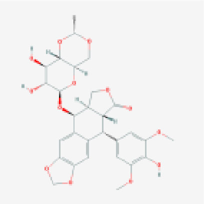

Etoposide

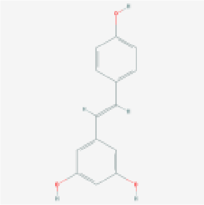

Resveratrol

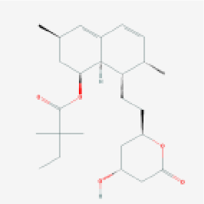

Simvastatin

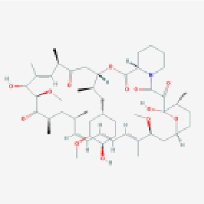

Sirolimus

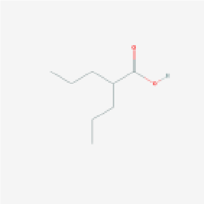

Valproic acid

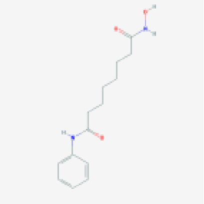

Vorinostat

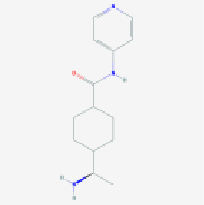

Y-27632

(b)

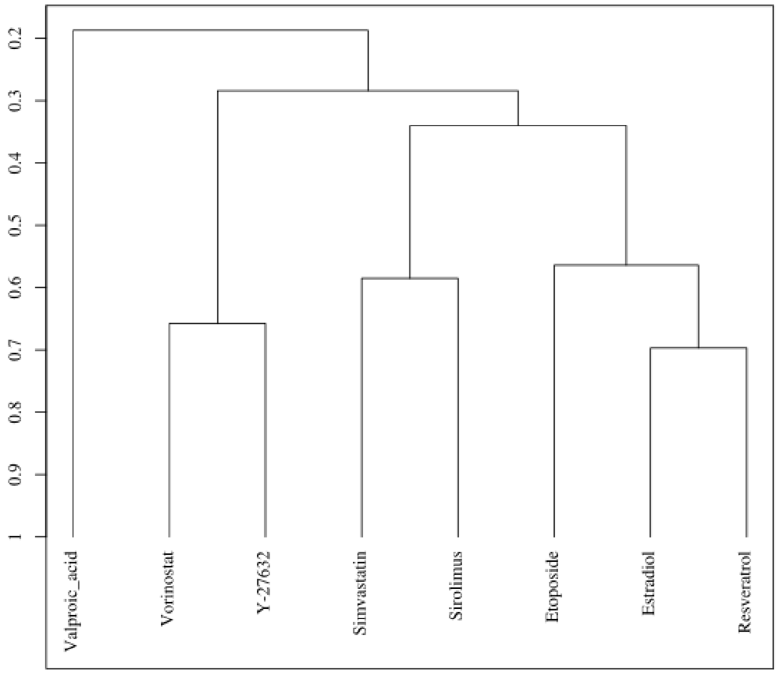

Supplementary Fig S6. Structures for the drug candidates identified multiple omics analysis. (a) The chemical structures of the eight drug candidates were shown. These structure were obtained in PubChem Project (https://pubchem.ncbi.nlm.nih.gov/). PubChem Compound Identifiers (CIDs) are follows; Estradiol (5757), Etoposide (36462), Resveratrol (445154), Simvastatin (54454), Sirolimus (5284616), Valproic acid (3121), Vorinostat (5311), Y-27632 (448042). (b) The dendrogram showed in the clustering results by the tanimoto similarity of chemical structures of the eight drug candidates. The structures were clustered by the complete linkage method. The vertical axis showed the value of tanimono coefficient.

Supplementary Table S1. The signature genes identified from GSE18090, GSE25226, and GSE38246. The 3,892 signature genes comprehensively obtained by collecting the signature genes ($p < 0.015$ in t-test) of each analysis. "U" and "D" indicate up-regulated and down-regulated genes, respectively.

Supplementary Table S2. The signature proteins detected from the proteomic analysis. The 389 signature proteins reported by proteomic analysis of Chiu et al. "U" and "D" indicate up-regulated and down-regulated genes, respectively.

Supplementary Table S3. Human protein and dengue viral protein interactions collected from the literature. The 268 human-virus PPIs collected from the literature were shown. The PPIs involved 10 dengue viral proteins and 221 human proteins. If the interaction found in literature, the interaction signed "T" that means true. We collected the union of human-viral protein interactions from literature.

Supplementary Table S4. The signature genes/proteins identified from the transcriptomic, proteomic and interactomic analyses. We listed the 3,892 signature genes identified by the transcriptomic analysis, the 389 signature proteins identified by the proteomic analysis, and the 221 human proteins identified by the human-virus PPIs. If the signature gene/protein identified in each omics analysis, the signature gene/protein assigned "T" that means true.

Supplementary Table S5. Common pathways identified from the transcriptome and proteome analyses. The 115 common pathways identified by GSEAs of the transcriptomic and proteomic data of dengue virus and human host pathways.

Pathway	P-value in Transcriptomic analysis	P-value in Proteomic analysis
BIOCARTA_CARM1_PATHWAY	9.36E-03	4.07E-02
BIOCARTA_CELLCYCLE_PATHWAY	1.28E-02	2.34E-03
BIOCARTA_DNAFRAGMENT_PATHWAY	1.19E-03	2.47E-02
BIOCARTA_G1_PATHWAY	7.56E-03	3.21E-02
BIOCARTA_G2_PATHWAY	5.69E-03	2.13E-02
BIOCARTA_PPARA_PATHWAY	2.53E-02	3.17E-03
BIOCARTA_RACCYCD_PATHWAY	9.94E-04	2.64E-02
BIOCARTA_RANMS_PATHWAY	1.14E-04	1.64E-03
BIOCARTA_RB_PATHWAY	9.36E-03	3.69E-03
BIOCARTA_RNA_PATHWAY	4.02E-02	2.47E-02
KEGG_CELL_CYCLE	1.43E-11	1.51E-02
KEGG_OOCYTE_MEIOSIS	1.49E-03	2.43E-02
KEGG_P53_SIGNALING_PATHWAY	1.12E-03	2.91E-02
KEGG_PROTEIN_EXPORT	1.83E-06	2.73E-04
PID_AURORA_A_PATHWAY	6.40E-04	9.46E-04
PID_AURORA_B_PATHWAY	6.62E-06	4.41E-05
PID_E2F_PATHWAY	4.02E-07	8.98E-05
PID_FOXM1_PATHWAY	1.12E-05	4.66E-07
PID_FOXO_PATHWAY	3.46E-03	3.38E-02
PID_IL1_PATHWAY	3.93E-02	9.89E-03
PID_MYC_ACTIV_PATHWAY	2.00E-08	3.92E-06
PID_P73PATHWAY	4.62E-03	4.79E-02
PID_PLK1_PATHWAY	2.79E-08	1.65E-06
PID_SMAD2_3NUCLEAR_PATHWAY	1.55E-02	3.56E-05
PWO_ANDROGEN_SIGNALING_PATHWAY	3.46E-02	6.84E-03
PWO_ANTINEOPLASTIC_AND_IMMUNOMODULATORY_DRUG_PATHWAY	2.70E-06	1.10E-05
PWO_ANTINEOPLASTIC_DRUG_PATHWAY	2.13E-06	1.02E-05
PWO_AVITAMINOSIS_DISEASE_PATHWAY	1.89E-02	2.88E-06
PWO_BRAIN_DISEASE_PATHWAY	9.23E-05	5.54E-04
PWO_CELL_CYCLE_PATHWAY	2.54E-12	1.26E-02
PWO_CELL_CYCLE_PATHWAY_MITOTIC	2.54E-12	1.26E-02
PWO_CHAPERONE_MEDIATED_AUTOPHAGY_PATHWAY	2.47E-02	3.50E-02
PWO_CLASSIC_METABOLIC_PATHWAY	1.95E-06	1.75E-03
PWO_CONGENITAL_DISEASE_PATHWAY	1.65E-05	4.60E-02
PWO_DIGESTIVE_SYSTEM_DISEASE_PATHWAY	9.82E-03	1.50E-03
PWO_DISEASE_PATHWAY	5.34E-10	1.68E-02
PWO_ETOPOSIDE_DRUG_PATHWAY	8.82E-04	3.34E-07
PWO_FOLATE_MEDIATED_ONE_CARBON_METABOLIC_PATHWAY	9.60E-04	1.90E-02
PWO_FOLIC_ACID_DEFICIENCY_PATHWAY	2.47E-02	3.50E-02
PWO_G1S_TRANSITION_PATHWAY	2.44E-03	1.97E-03
PWO_G2M_CHECKPOINT_PATHWAY	4.43E-02	6.26E-04
PWO_GASTROINTESTINAL_DISEASE_PATHWAY	1.09E-04	1.90E-03
PWO_GEMCITABINE_PATHWAY	9.36E-03	4.07E-02
PWO_INBORN_ERROR_BRAIN_METABOLIC_DISEASE_PATHWAY	1.11E-03	3.02E-03
PWO_INBORN_ERROR_METABOLISM_DISEASE_PATHWAY	1.65E-05	4.60E-02
PWO_INBORN_GENETIC_DISEASE_PATHWAY	1.65E-05	4.60E-02
PWO_INTERLEUKIN_1_FAMILY_MEDIATED_SIGNALING_PATHWAY	5.11E-03	2.96E-02
PWO_INTESTINAL_DISEASE_PATHWAY	1.09E-04	1.90E-03
PWO_IRINOTECAN_DRUG_PATHWAY	1.79E-02	1.37E-06
PWO_MALABSORPTION_SYNDROME_PATHWAY	2.47E-02	3.50E-02
PWO_METHOTREXATE_DRUG_PATHWAY	2.47E-02	3.50E-02
PWO_MITOCHONDRIAL_ENCEPHALOMYOPATHY_PATHWAY	1.55E-03	2.13E-02
PWO_NERVOUS_SYSTEM_DISEASE_PATHWAY	1.11E-12	7.17E-04
PWO_NUTRITIONAL_AND_METABOLIC_DISEASE_PATHWAY	1.73E-05	2.15E-03
PWO_NUTRITIONAL_DISORDER_PATHWAY	2.53E-02	1.23E-05
PWO_OROTIC_ACIDURIA_DISEASE_PATHWAY	1.55E-03	2.13E-02
PWO_P53_SIGNALING_PATHWAY	1.47E-02	1.86E-02
PWO_PATHWAY_PERTINENT_TO_DNA_REPLICATION_AND_REPAIR_CELL_CYCLE_MAINTENANCE_OF_GENOMIC_INTEGRITY_RNA_AND_PROTEIN_BIOSYNTHESIS	3.86E-11	1.49E-02
PWO_PATHWAY_PERTINENT_TO_PROTEIN_FOLDING_SORTING_MODIFICATION_TRANSLOCATION_AND_DEGRADATION	3.46E-17	2.82E-05
PWO_PROTEIN_DEGRADATION_PATHWAY	1.88E-17	2.57E-05
PWO_REGULATORY_PATHWAY	4.89E-20	3.51E-05
PWO_SEX_STEROIDS_SIGNALING_PATHWAY	3.12E-02	4.88E-02
PWO_SORAFENIB_DRUG_PATHWAY	2.15E-03	1.51E-06
PWO_TRANSCRIPTION_FACTOR_MEDIATED_SIGNALING_PATHWAY	4.75E-02	4.22E-02
PWO_TRANSCRIPTION_PATHWAY	2.46E-02	3.10E-02
PWO_TRANSCRIPTION_PATHWAY_VIA_TRANSCRIPTION_FACTOR_MEDIATED_SIGNALING	4.75E-02	4.22E-02
PWO_UROGENITAL_DISEASE_PATHWAY	3.76E-02	4.97E-02
PWO_UROLOGIC_DISEASE_PATHWAY	3.76E-02	4.97E-02
PWO_X_LINKED_GENETIC_DISEASE_PATHWAY	1.20E-02	2.11E-03
REACTOME_ACTIVATED_TLR4_SIGNALLING	2.49E-02	2.91E-02
REACTOME_ACTIVATION_OF_CHAPERONE_GENES_BY_XBP1S	6.96E-07	2.76E-02
REACTOME_ADAPTIVE_IMMUNE_SYSTEM	1.55E-07	2.94E-02
REACTOME_ANTIGEN_PRESENTATION_FOLDING_ASSEMBLY_AND_PEPTIDE_LOADING_OF_CLASS_I_MHC	1.99E-02	1.65E-03
REACTOME_ANTIGEN_PROCESSING_CROSS_PRESENTATION	2.63E-07	4.16E-02
REACTOME_ANTIVIRAL_MECHANISM_BY_IFN_STIMULATED_GENES	1.27E-03	6.94E-08
REACTOME_APC_C_CDC20_MEDIATED_DEGRADATION_OF_MITOTIC_PROTEINS	1.07E-09	3.59E-02
REACTOME_APC_C_CDH1_MEDIATED_DEGRADATION_OF_CDC20_AND_OTHER_APC_C_CDH1_TARGETED_PROTEINS_IN_LATE_MITOSIS_EARLY_G1	1.16E-08	9.17E-03
REACTOME_ASPARAGINE_N_LINKED_GLYCOSYLATION	1.95E-10	1.58E-02
REACTOME_CALNEXIN_CALRETICULIN_CYCLE	1.50E-02	2.96E-02
REACTOME_CELL_CYCLE	4.78E-33	6.46E-06
REACTOME_CELL_CYCLE_CHECKPOINTS	9.37E-12	3.62E-02
REACTOME_CELL_CYCLE_MITOTIC	1.24E-27	6.63E-06
REACTOME_CYCLIN_A_B1_ASSOCIATED_EVENTS_DURING_G2_M_TRANSITION	5.88E-03	5.66E-03
REACTOME_CYTOKINE_SIGNALING_IN_IMMUNE_SYSTEM	2.86E-04	4.42E-04
REACTOME_DIABETES_PATHWAYS	3.99E-04	1.86E-02
REACTOME_DNA_REPLICATION	1.79E-28	9.37E-05
REACTOME_E2F_MEDIATED_REGULATION_OF_DNA_REPLICATION	6.80E-05	1.67E-03
REACTOME_FATTY_ACID_TRIACYLGLYCEROL_AND_KETONE_BODY_METABOLISM	2.94E-02	9.64E-03
REACTOME_G1_S_SPECIFIC_TRANSCRIPTION	8.35E-05	1.12E-02
REACTOME_G1_S_TRANSITION	3.40E-19	7.03E-03
REACTOME_G2_M_CHECKPOINTS	3.00E-05	2.57E-02
REACTOME_HIV_INFECTION	1.33E-06	3.55E-02
REACTOME_HIV_LIFE_CYCLE	3.87E-02	1.32E-02
REACTOME_HOST_INTERACTIONS_OF_HIV_FACTORS	6.42E-06	5.89E-03
REACTOME_IMMUNE_SYSTEM	3.19E-09	5.61E-05
REACTOME_INTERFERON_SIGNALING	3.78E-04	4.40E-05
REACTOME_KINESINS	1.79E-02	1.37E-06
REACTOME_METABOLISM_OF_NON_CODING_RNA	1.32E-03	1.99E-04
REACTOME_METABOLISM_OF_PROTEINS	7.65E-08	2.00E-02
REACTOME_MHC_CLASS_II_ANTIGEN_PRESENTATION	2.53E-04	4.40E-04
REACTOME_MITOTIC_G1_G1_S_PHASES	6.81E-19	7.47E-03
REACTOME_MITOTIC_M_M_G1_PHASES	1.62E-24	1.04E-04
REACTOME_MITOTIC_PROMETAPHASE	5.45E-11	1.47E-03
REACTOME_MRNA_PROCESSING	1.64E-05	4.95E-02
REACTOME_MYD88_MAL_CASCADE_INITIATED_ON_PLASMA_MEMBRANE	1.82E-02	1.77E-02
REACTOME_N_GLYCAN_TRIMMING_IN_THE_ER_AND_CALNEXIN_CALRETICULIN_CYCLE	9.36E-03	3.69E-03
REACTOME_POST_TRANSLATIONAL_PROTEIN_MODIFICATION	5.34E-04	4.69E-02
REACTOME_PROCESSING_OF_CAPPED_INTRON_CONTAINING_PRE_MRNA	5.83E-07	2.44E-02
REACTOME_RECRUITMENT_OF_NUMA_TO_MITOTIC_CENTROSOMES	4.02E-02	1.64E-03
REACTOME_REGULATION_OF_MITOTIC_CELL_CYCLE	8.25E-13	2.63E-04
REACTOME_TOLL_RECEPTOR_CASCADES	1.68E-02	9.52E-03
REACTOME_TRAF6_MEDIATED_INDUCTION_OF_NFKB_AND_MAP_KINASES_UPON_TLR7_8_OR_9_ACTIVATION	4.29E-02	1.25E-02
REACTOME_TRANSPORT_OF_MATURE_MRNA_DERIVED_FROM_AN_INTRONLESS_TRANSCRIPT	3.12E-02	1.39E-05
REACTOME_UNFOLDED_PROTEIN_RESPONSE	3.83E-07	8.48E-04
SA_REG_CASCADE_OF_CYCLIN_EXPR	3.79E-02	4.07E-02
